# Socio-political and organizational influences on national infectious disease surveillance for refugees: A qualitative case study in Lebanon

**DOI:** 10.1371/journal.pgph.0001753

**Published:** 2023-06-12

**Authors:** Majd Saleh, Natasha Howard

**Affiliations:** 1 Department of Global Health & Development, London School of Hygiene & Tropical Medicine, London, United Kingdom; 2 Saw Swee Hock School of Public Health, National University of Singapore and National University Health System, Singapore, Singapore; New York University Grossman School of Medicine, UNITED STATES

## Abstract

Infectious disease surveillance provides actionable information on displaced populations and helps identify outbreaks. Though not a signatory to the 1951 Refugee Convention, Lebanon has experienced large refugee influxes (e.g. Palestinians in 1948, Syrians in 2011), yet information on socio-political and organizational influences shaping surveillance targeting refugees is limited. We thus aimed to examine how Lebanese socio-politics affected infectious disease surveillance for refugees in Lebanon. We conducted a *qualitative multimethod single* case study of government engagement with refugee infectious disease surveillance (2011–2018) drawing from document analysis, semi-structured observations, and semi-structured key informant interviews at four surveillance sites in Lebanon. We analysed data thematically, using deductive and inductive coding. National politics delayed government and thus its epidemiological surveillance program’s (ESU) engagement with refugee disease surveillance, largely due to Lebanon not being a 1951 Refugee Convention signatory and internal policy disagreements. Thus, it was initially difficult for the ESU to lead surveillance activities, though it later became more active. The ESU was limited by unclear reporting mechanisms and resources and its reliance on aggregated surveillance data prevented provision of data-informed responses. Though the ESU led surveillance nationally, and we identified positive provincial level collaborations due to individual efforts, some partners still conducted parallel surveillance. We found no systematic approach to infectious disease surveillance for refugees. The ESU could improve surveillance for refugees by collaborative strategic planning with partners for preparedness, surveillance, reporting, and sustainable resource allocation during refugee crises. Further suggestions include collecting disaggregated data, and piloting potentially more efficient syndromic surveillance, based on symptom clusters, for refugee populations.

## Introduction

Lebanon has long hosted displaced populations from neighbouring countries, primarily from Palestine and Syria but also countries such as Iraq and Sudan [1,2]. However, Lebanon remains ambivalent about refugees. It has not signed the 1951 United Nations Convention Relating to the Status of Refugees [2,3], and its political fragmentation and indecision on refugee issues is well documented [4,5]. Lebanon’s representatives expressed uncertainty about the Convention’s obligations, especially towards Palestinians in Lebanon, referring to forced migrants as neighbouring national ‘guests’ rather than ‘refugees’ and preferring third parties care for them [2].

Lebanon’s first recorded mass cross-border inflow was approximately 110,000 Palestinians after the 1948 *Nakba* [Arabic for catastrophe or ethnic cleansing][4]. The second major displacement began from the 2011 start of the Syrian conflict, with over 1.5 million Syrians fleeing to Lebanon—the highest number per capita in the world [2]. As Lebanon’s parliament and political parties disagreed on how to respond from the beginning of the Syrian conflict-related displacement, no decision was made [6]. While refugee numbers have decreased, recent estimates indicate 174,422 Palestinians as of 2017 Lebanese census [7], and 814,715 registered Syrian refugees as of 2022 United Nations High Commission for Refugees (UNHCR) report [8]. Both populations have restricted civil rights [4,9] and live in substandard informal urban or tented settlements [4,5], often lacking safe water and sanitation [6].

Camps and settlements of forcibly-displaced people can be overcrowded and often lack basic needs, e.g. water, sanitary supplies, adequate nutrition, and environmental protection [10–12]. In displacement settlements, infectious disease surveillance is important to enable ongoing information for action and to identify outbreaks to initiate immediate interventions [10–12]. However, literature on infectious disease surveillance targeting refugees is minimal, particularly in West Asia, despite the region’s high prevalence of displaced and refugee populations [13,14].

Infectious disease surveillance and mandatory reporting in Lebanon began in 1957 [15]. The Epidemiological surveillance programme (ESU), hosted at the Ministry of Public Health (MOPH) headquarters in Beirut is responsible for implementing this law and monitors 40 infectious diseases and syndromes plus cancer data [16]. ESU’s additional core functions are to screen epidemiological alerts, detect and investigate outbreaks, train and sensitise on surveillance methods, and disseminate health information [17]. Despite its importance, there is virtually no academic literature on ESU organisational aspects or information related to its core functions.

Additionally, surveillance programs cannot operate alone. The World Health Organization (WHO) and surveillance literature identify collaboration with response actors and affected communities (e.g. representatives of displaced/refugee populations) as crucial for effective and comprehensive surveillance during crises, such as the displacement of Syrian refugees post-2011 [12,14]. Collaborations are important, especially with laboratory diagnostic centres, vaccination providers, international funders and implementing organisations, and affected communities [12,18,19]. We found, however, no research examining socio-political or organizational (e.g. MOPH, ESU) contexts affecting infectious disease surveillance in Lebanon, or how these shaped collaborative activities between surveillance programme, other response actors, or refugees.

This study thus aimed to examine how national socio-politics and internal ESU organisational factors affected infectious disease surveillance for refugees in Lebanon. Objectives were to: (i) identify the ways in which national context influenced infectious disease surveillance for refugees; (ii) examine additional influences of internal organisational factors; and (iii) identify lessons to improve infectious disease surveillance for refugees in Lebanon and potentially elsewhere.

## Methods

### Study design

We conducted a qualitative multimethod single case study of the ESU’s infectious disease surveillance for refugees, drawing from literature [14] document reviews, key informant interviews, and semi-structured observations conducted at four study sites in 2020: (i) the ESU central office in Beirut; (ii) ESU Bekaa provincial office; (iii) a mobile medical clinic at an informal tented settlement for Syrians; and (iv) a United Nations Relief and Works Agency (UNRWA) health facility for Palestinian refugees. Case studies enable examination of an event or organization in situ, without changing its characteristics, while multimethod approaches allow between-method triangulation that improves credibility and transferability [20–22].

Our research question was: “How *have socio-political factors*, *internal and external to the ESU*, *affected refugee infectious disease surveillance in Lebanon*?”

### Data collection

#### Document review

MS purposively searched: (i) publicly available online documentation on infectious disease surveillance, including laws and legislative decrees of the Lebanese Parliament 1900–2019, MOPH memos and circulars, and WHO assessments; and (ii) internal ESU documentation, including policy documents, circulars, official letters, guidelines, and constitutions, accessed June-November 2018 in the archives at ESU headquarters and Bekaa provincial offices, with permission from the ESU director. Documents that helped answer research questions were selected, scanned, and saved in a password-protected folder only accessible to investigators. MS extracted data using the following headings: publication date, title, document type and whether ESU/internal or external, topic or activities described.

#### Key informant interviews

We developed a semi-structured interview guide, informed by a literature review [14] and senior ESU staff. MS recruited interviewees purposively based on their involvement in refugee health in Lebanon and knowledge of refugee and migrant policies, surveillance activities, and the ESU. MS contacted 24 potential interviewees in Arabic and English by email or phone, two of whom did not respond and two declined. Interviews took on average 40 minutes and were audio recorded after providing informed written consent. Two interviewees preferred answering questions through email and three preferred no audio-recording. MS conducted and transcribed interviews in English, except one conducted in Arabic and translated and transcribed into English. We ensured interviewee confidentiality by conducting interviews in times and locations of interviewees’ choosing. To ensure interviewee anonymity, no names or personal data were requested in audio recordings and MS assigned interviewees a numerical ID code for audio recordings, transcripts, and study outputs.

#### Observations

We drafted a semi-structured observation guide, based on literature findings and expert feedback, which we amended iteratively. MS prepared fieldnotes describing ten days of observations conducted in our four sites, to help interpret interview findings and determine how documentation was implemented in practice, as observation could help identify elements of behaviour and environment not captured through interviews [22].

### Analysis

We analysed document, transcript, and fieldnote data thematically in NVivo 12-plus software using deductive and inductive coding [23,24]. In summary, MS conducted data familiarisation during transcription, then generated initial codes, and collated potential themes. MS and NH then reviewed, defined, and named themes through iterative discussion. Broad deductive themes included political and socioeconomic factors, ESU internal factors, and refugee surveillance activities, with inductive subthemes generated from collating and interpreting codes within deductive themes [24]. We determined data saturation when no new thematic refinements were identified from data, while acknowledging Braun & Clark’s stance that saturation is conceptually problematic [22,24,25].

We categorised institutional stakeholders working with ESU on surveillance related to refugees [26] in terms of: (i) Actor involvement, categorised as supportive, neutral, or competitive; (ii) Actor interest in surveillance activities, categorised as high, medium, or low; and (iii) Actor influence on surveillance activities, categorised as high, medium, or low.

### Reflexivity

MS is a former ESU surveillance coordinator, trained as an epidemiologist, conducting this study as part of her doctoral research. While refugee disease surveillance was unrelated to her work, her insider perspective on ESU influenced study design, data collection, and interpretations. NH is her supervisor, and an experienced social scientist, who helped interrogate the research process to improve rigour and transparency.

### Ethics

We obtained ethics approval from the London School of Hygiene & Tropical Medicine Observational Research Ethics Committee in the UK (reference 15463) and Rafik Hariri University Hospital ethics board in Lebanon (Saleh, M 11/06/18), and permission of the Lebanese MOPH Director General (reference 14605/1/18).

## Results

### Source characteristics

Table 1 shows 50 sources (i.e. 20 document, 20 interview, and 10 observation**)** used in our analysis. Given the small number of key informants on infectious disease surveillance in Lebanon are known to each other, we refrained from reporting interviewee characteristics to protect anonymity.

**Table 1:** Data sources and numbers of documents, interviewees, and observations.

**Document type**	**Topics**	**Documents (n = 20)**
Legislative decrees	MOPH organizational structure	2
National laws	Infectious disease identification and reporting from health facilities to MOPH	3
MOPH circulars, memos, official letters	Addressing health facilities on surveillance amendments, trainings, reporting, and support	9
MOPH decisions	ESU duties	1
ESU SOPs and guidelines	Standard surveillance procedures and guidance	3
WHO assessment reports	Internal reports on WHO surveillance assessment	2
**Interviewee type**	**Identification code**	**Interviewees (n = 20)**
MOPH	MOPH 01–04	4
ESU	ESU 01–03	3
United Nations agencies	UN 01–05	5
INGO/NGO	NGO 01–02	2
Government hospitals	GH 01–02	2
Medical centres	MC 01–02	2
Researchers and analysts	RA 01–02	2
**Fieldnote type**	**Identification code**	**Observations (n = 10)**
Central ESU office	FN1	5
Peripheral ESU office	FN2	2
Mobile medical unit	FN2	1
Informal tented settlement	FN2	1
UNRWA health facility	FN3	1

Fieldnote 1 describes five observations of staff activities in ESU headquarters. Fieldnote 2 describes four observation days during a visit to Bekaa, the province with the most informal refugee settlements [27], i.e. 2 on daily activities of a surveillance coordinator/epidemiologist working on refugee infectious disease surveillance, 1 of a mobile medical unit, and 1 in a Syrian informal tented settlement of how data are recorded and reported. Fieldnote 3 describes a day observing data recording and reporting activities at a UNRWA health facility in Chatila Palestinian refugee camp in Mount Lebanon Province.

We organised findings under three themes: (i) national politics affecting refugee surveillance; (ii) ESU organisational structure affecting surveillance delivery; and (iii) refugee surveillance collaboration.

### Politics and refugee surveillance

Table 2 shows political and socio-economic enablers and barriers to infectious disease surveillance for refugees. We identified three inductive sub-themes that each affected infectious disease surveillance for refugees in Lebanon: (i) not being a 1951 Refugee Convention signatory; (ii) slow adaptive response to Syrian conflict; and (iii) ESU’s initial invisibility. Table 2 shows political and socioeconomic enablers and barriers to infectious disease surveillance for refugees.

**Table 2:** Political and socioeconomic enablers and barriers to national infectious disease surveillance for displaced populations in Lebanon.

**Political enablers**	**Political barriers**
• Lebanon is a signatory of several UN conventions. • National commitment and support for universal health coverage including support for primary healthcare services, which should in principle include refugees.	• Lebanon is not a 1951 Refugee Convention signatory. • External and internal political conflicts. • Sectarian governmental system. • Weak governance since the civil conflict (1975–1990) • Disagreement between political parties on a response to displaced Syrians. • Slow government involvement in all refugee crises.
**Socio-economic enablers**	**Socio-economic barriers**
• International technical and financial support for refugee responses, with some international funding continuing. • Eventual development of a national strategy (response plan) in 2017. • Coordination with stakeholders. • National transition to electronic databases for online reporting and health information. • Faxes were still used, helping with archiving. • Motivated personnel at peripheral level.	• Large influxes of forcibly-displaced populations. • Market-driven healthcare system. • Refugees residing in informal tented settlement and within host communities, so sometimes difficult to identify. • Lack of enumeration of displaced populations. • Donor fatigue and reductions in international funding for displaced Syrians and Palestinians. • Weak national coordination and prioritisation of funding. • Lack of human resources at district levels • Poor maintenance and technical issues with electronic databases. • Unreliable electricity and unexpected outages.

#### Consequences of not being a Refugee Convention signatory

A dominant issue for interviewees was that Lebanon had not signed the 1951 Refugee Convention and had no laws relating to refugees [2,3] (UN-01, UN-05, ESU-03, NGO-03). Interviews aligned with Janmyr’s analysis [2] that Lebanese representatives were uncertain about the Convention’s obligations, preferring to call refugees ‘guests’ and having third parties care for them [2]. Signing the convention would give the Lebanese government a set of obligations towards refugees, rather than responding out of hospitality or charity [2,28]. Despite international efforts to have Lebanon sign the convention, ongoing national political turmoil has made the Convention a relatively low political priority [2]. Most interviewees reported that consequently Lebanon officially refers to refugees as “*displaced persons*” [2] (ESU-03, NGO-03). Several described how, at the start of the Syrian conflict, Lebanese parliamentarians disagreed about how to respond. One party welcomed Syrian refugees and wanted official refugee camps and the other opposing political party refused [5,29] (NGO-03, RA-02). As a result, refugees either rented private accommodation or organised informal tented settlements (ITS) while receiving assistance from UN agencies and international non-governmental organisations (INGOs) [5,29] (NGO-03, RA-02).

#### Slow adaptive response to Syrian conflict

Interviewees noted that government indecision on how to respond to mass displacement from the Syrian conflict was initially manageable, but as refugee numbers increased to around one million in 2013, with no national strategy setting clear terms, the government, including ESU had to respond [30] (NGO-03, RA-02, ESU-02). Some interviewees described this process as slow and late (ESU-02, NGO-03, RA-02), with one characterising involvement as an *“adaptive process*" (RA-02).

According to some interviewees, ESU’s work with refugees mirrored the government’s slow engagement (ESU-02, NGO-3). From 2011 until 2013, the ESU was similarly unengaged and did not actively monitor infectious diseases in displaced communities (ESU-02, MOPH-04, NGO-3). Instead, several claimed the lack of disaggregation in indicator-based surveillance meant that the ESU’s monitoring treated everyone indistinguishably despite differing risks (ESU-02, MOPH-04). After the 2013 increase in refugee numbers, some aspects of ESU surveillance changed. For example, due to potential outbreaks, some systems were adapted and presumably included most Syrian refugees in Lebanon (ESU-01-03, NGO-01).

Some interviewees described the lack of government policy on establishing official refugee camps as having made surveillance harder in terms of identifying refugee populations and estimating their true numbers (ESU-02-03). Many MOPH interviewees indicated that UNHCR numbers only reflect registered refugees and not the true numbers integrated within communities (ESU-01, ESU-02, ESU-03). Thus, surveillance could have been easier if refugee camps existed (NGO-03).

#### ESU’s initial invisibility

Due to ESU’s slow engagement in the refugee crisis, and despite its years of work with international organizations in Lebanon, interviewees reported that many did not know of its existence (ESU-02, NGO-03). Two noted that some INGOs called for development of an infectious disease surveillance system for refugees, before discovering that ESU’s surveillance was already in place (ESU-02, NGO-03). These interviewees contributed to cessation of this parallel surveillance initiative. NGO-03 recounted how, once introduced to the ESU team, she pushed for international support of the national surveillance system (NGO-03). ESU-02 considered it a personal initiative to attend UNHCR-led health working-group meetings, which were not part of her job description (ESU-02). Both interviewees described how the ESU was recognised after 2013, and took on leadership of infectious disease surveillance in Lebanon’s refugee response (ESU-02, NGO-03):

*"The first time I went there [health working group meeting], they said, *’Oh there is someone coming from the ministry of health*!’ […] At that time people wanted to do a new surveillance system. They didn’t know we already had a surveillance system […] Now they rely on our data." ESU-02*.

### Organisational factors affecting surveillance activities

**Fig 1:**
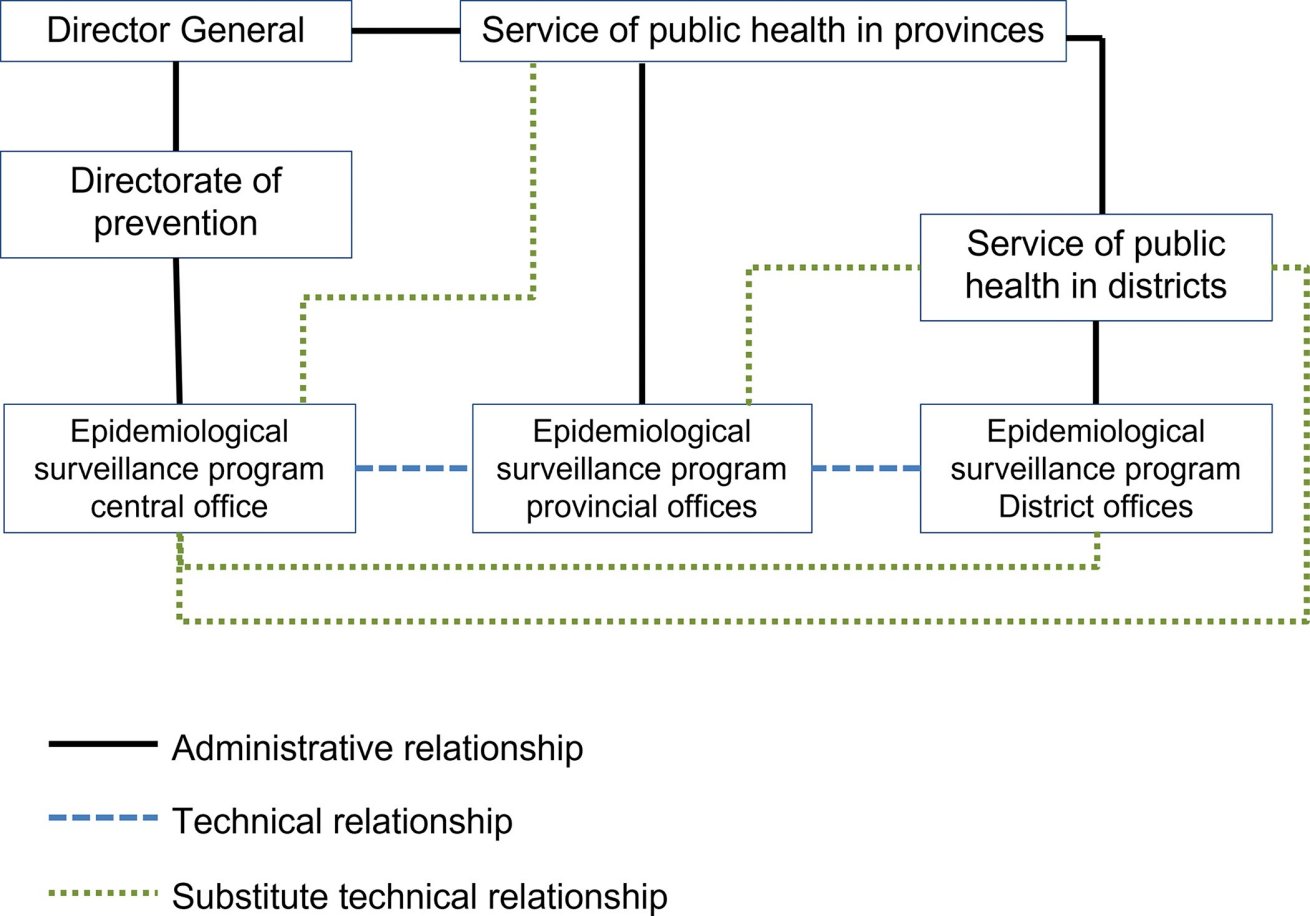
Possible ESU hierarchy based on documents and interviews.

We identified three inductive sub-themes affecting ESU’s work: (i) ESU mandate and unclear hierarchy; (ii) decision-making and reporting; and (iii) national surveillance adaptations for refugees. Fig 1 provides the ESU organogram we developed based on findings.

#### ESU mandate and unclear hierarchy

ESU’s mandate was based on the 1957 Governmental Law, listing notifiable diseases that must be reported by treating physicians and health facilities [15] (FN-01; ESU-01, ESU-02). This law remains unchanged, but the list of diseases is amended as necessary and shared with reporting facilities through circulars (Governmental Law on reportable disease, 1957; FN-01; ESU-03), with the last amendment in MOPH decision #1/899 in 2014 adding Leishmaniasis. ESU’s objectives were to measure disease burden and detect outbreaks, so it was primarily tasked with infectious disease surveillance (MOPH-01, MOPH-04, ESU-03). However, cancer surveillance was an ESU responsibility added after it was unsuccessfully coordinated elsewhere in the MOPH [17] (ESU-03). Additionally, the ESU was not responsible for tuberculosis, HIV, or malaria surveillance, which were implemented by a separate surveillance programme for reasons potentially related to external funding (ESU-03; FN-1). Several interviewees noted that since inception, control centres for these three diseases primarily provided treatment and only secondarily collected and analysed data [31] (MOPH-01, MOPH-04, ESU-03).

Within the MOPH, the Director General was responsible for directorates of medical care, central public health laboratory, provincial public health services, and preventive healthcare [32]. The ESU was established in 1995 as a unit within the directorate of preventive healthcare and expanded into a national programme in 2005 but was never integrated within the official MOPH organisational structure (FN-01). Like the MOPH, the ESU has a dual hierarchy and reporting structure (FN-02; ESU-03), with the central ESU office in Beirut supervising peripheral ESU offices in provinces and districts (MOPH decision #1/80, 2018; FN-01, FN-02; ESU-03) while the peripheral public health services headed by a peripheral physician supervises ESU peripheral teams (MOPH decision #1/80, 2018; FN-01, FN-02; ESU-02, ESU-03).

No official ESU organogram existed to explain this dual supervision structure (FN-01, FN-02), but Fig 1 provides our interpretation based on interview and document evidence. The ESU central office reports to the DG, while provincial ESU teams report to both the provincial public health services and ESU central office and district teams report to both district public health services and ESU provincial office. Despite decisions and circulars mentioning the ESU team duties, interviewees described a lack of official terms of reference for ESU personnel or partners working on surveillance. Especially at the periphery, work relationships were based on personal efforts (FN-01, FN-02; ESU-01, ESU-02, GH-01). Several interviewees expressed uncertainty about reporting to multiple people (FN-02; ESU-02). “There is nothing written, like to who I should report, to the district physician or to the province physician” (ESU-02).

#### ESU decision-making and reporting

The ESU lacked mechanisms to formally record and disseminate internal decisions, with some interviewees noting that no mandates existed for what ESU personnel could or could not do, with decisions communicated ad hoc by phone, social media, or face-to-face (FN-01; ESU-01, ESU-02). Interviewees described instances of internal ESU decisions occurring without official documentation, instead relying on *bouche-à-oreille* (word-of-mouth) (ESU-02), especially during the Syrian refugee displacement (MOPH-01, MOPH-03). For example, decisions on surveillance system adaptation were only shared orally during meetings (FN-01; ESU-01). This engendered mixed feelings, with some interviewees indicating the importance of documenting decisions, while others noted that everyone within the team would eventually be aware: “It is mainly because who is working with those measurements are part of our team, so we do not need those circulars” (ESU-01).

For disease identification and reporting, ESU interviewees described its two systems as: (1) indicator-based and (2) event-based (ESU-02, ESU-03). Indicator-based routine surveillance, was described by interviewees and documentation as a passive mode of surveillance requiring immediate or weekly reporting [15]. Data are reported from different reporting sites by physicians, nurses, or administration personnel at hospitals, medical centres (i.e. primary healthcare centres, dispensaries), laboratories, and schools [17] (ESU-02, ESU-03). Event-based surveillance, or rumour detection, in comparison, can be obtained from the public via MOPH hotline, social media, or ESU email (FN-01; ESU-03). Interviewees and observations indicated routine surveillance reporting is done through fax, though this has slowly transitioned to online reporting (FN-01; ESU-02, ESU-03). This online platform is used for very few programmes and periodically suffers from technical issues (FN-01; ESU-03).

#### National surveillance adaptations for refugees

The ESU provided no surveillance specific to refugees, with the MOPH interviewees indicating no disaggregation between Lebanese and non-Lebanese in ESU work or guidelines (ESU-02, ESU-03). While the UNRWA operated its own surveillance for Palestinians, Syrians were described as integrated within the Lebanese population and using the same healthcare services so they would be captured in passive reporting eventually (ESU-01, ESU-02, ESU-03). Standard ESU reporting forms began including ‘Nationality’ to stratify Syrian refugees during analysis, but still did not include other refugees such as Palestinians, and this proxy did not indicate whether they were actually refugees or pre-conflict residents [33] (FN-01; ESU-02).

Other ESU surveillance systems were adapted to include Syrian refugees. For example, the ESU Acute Flaccid Paralysis (AFP) surveillance team adapted activities to find Syrian refugees after the 2013 polio outbreak in Syria, considering any Syrian AFP case as highly suspected for polio (i.e. a "hot case") and taking additional measures including intensified hospital visits, specimen collection from family members, and vaccination in case residence area [34,35] (Official Letter on hospital active surveillance for AFP, 2014; ESU-01). Similarly, increased *Viral Hepatitis A* (*VHA*) cases among refugees in 2013 [33,36](ESU-02) led to the creation of a specific *VHA* reporting form that included refugee status (i.e. refugee, non-refugee) and location (house, informal settlement) to speed detection (MOPH circular 15, 2015; ESU-02). A relatively unsuccessful adaptation was leishmaniasis reporting [37,38] (GH-02). After it was added to the standard reporting form, 28 leishmania treatment centres were established at ten government hospitals throughout Lebanon (ESU-02, GH-02) to identify and report cases to ESU (ESU-02). However, interviewees indicated this surveillance was dormant as reporting forms were not collected routinely and official numbers were outdated (FN-01; GH-02; ESU-02). The reason, according to an interviewee, was the absence of an assigned epidemiologist to follow up reports (ESU-02).

The main non-ESU surveillance initiatives that included refugees were the Lebanese National Tuberculosis Program (NTP), the National AIDS Program (NAP), and the UNRWA for Palestinians. The NTP had nine tuberculosis centres throughout Lebanon collecting data and conducting active screening and case finding in ITS and collective shelters for refugees (MOPH-01). The NAP coordinated HIV surveillance with partners, linking tests to treatment for ‘all nationalities residing in Lebanon’ (MOPH-04). The UNRWA, established in 1948 to assist displaced Palestinians, remained the primary contributor to Palestinian healthcare [4,39] with well-functioning infectious disease surveillance for Palestinians in Lebanon that paralleled ESU efforts (FN-03; UN-01, UN-02).

### Refugee surveillance collaboration

We identified two inductive sub-themes influencing refugee surveillance coordination and communication: (i) intragovernmental collaboration; (ii) stakeholder coordination and communication. Table 3 provides a stakeholder analysis of ESU’s potential collaborating partners.

**Table 3:** Stakeholder analysis.

Main Stakeholders	Characteristics				
	Involvement in refugee crisis	Interest in refugee surveillance	Influence/power on refugee surveillance activities	Collaborative Position with ESU	Impact of ESU surveillance on actor
Private academic research institutes	Evidence-based research, collaborate in projects, provide laboratory support as reference laboratories, reporting partners	High: Mostly for research and consultation purposes	High: Media and population rely on many of the findings of research centres	Neutral-Supportive	Low: Very independent, self-sufficient, do not rely on ESU information since they generate their own
UNHCR	Protection agency- leading coordination of the crisis in Lebanon	High: For suggesting programs related to refugees other than Palestinian	High: OVs & (I)NGOs report to them—coordinate all agencies working with refugees	Supportive: report and collaborate with ESU, follow up on ITS infectious disease investigation	High: In terms of infectious diseases, ESU provides UNHCR with information needed for follow-up
WHO	Funding and technical assistance, sometimes reporting partners	High: For suggesting programs	High: binding international regulations such as international health regulations (IHR)	Neutral-Supportive: depending on personnel at local office	High: WHO support is reflected in the outcomes of MOPH-ESU
UNRWA	Follow up on Palestinian refugees	High: working exclusively with Palestinian refugees	High: their presence lessens burden on Lebanese government, parallel surveillance system	Neutral-Supportive	Low: have a parallel disease surveillance system in place for Palestinians, do not rely very much on ESU data
UNICEF	Support EPI in vaccination	High: support all refugees	Neutral	Neutral	High: their support is reflected in health outcomes, especially vaccinations
(I)NGOs	Provide health services and important reporting partners	High: forefront for receiving refugees, established MMUs	High: presence in field makes them well-rounded with reality of the crises	Neutral-supportive: need to be better involved	Medium: (I)NGOs support PHCs receiving refugees—good source of information
Health facilities/reporting sites	Provide health services and important reporting partners	Medium: not all are concerned with refugees	Medium—High: in terms of finding and reporting IDs	Neutral—Supportive: some abide by 1957 law	High-medium: They need to be certified by MOPH to be able to practice, feel pressure to report
Directorate of prevention	Administratively supervisor of ESU, program support	High	High	Supportive	High: administratively involved
PHC service	Provide health services and important reporting partners	Medium	Low	Neutral	High: Improvement of reporting at PHC can improve overall data completeness for ESU
EPI	Ensure proper vaccination coverage	High: ESU eyes of EPI	Low	Supportive	High: ESU eyes of EPI
Director general of MOPH	Program support, direct supervisor of district health services	High: surveillance fits into Universal Health Coverage (UHC) spectrum	High: main decision-making role	Supportive	High: surveillance important component for UHC
Preventive medicine service	Ensure prevention and control of IDs	High: data informs programs	Neutral	Neutral	High: ESU numbers reflect programs

#### Intragovernmental collaboration

Internal collaboration between the MOPH departments varied considerably, as an ESU interviewee noted: "I think […] we have good collaboration with people outside the ministry. I think coordination with people or departments within the Ministry of Health, this is lacking…" (ESU-02). An important MOPH partner working with ESU was the expanded program on immunization (EPI) (FN-01; MOPH-03, NGO-03). The ESU was described as the “eyes of the EPI" (ESU-01, MOPH-03), as it communicated vaccine preventable disease (VPD) findings to the EPI (FN-01; ESU-01, MOPH-03). Successful collaboration between the ESU and the EPI can be partially explained by the fact that most VPD are managed vertically within the ESU by proactive individual epidemiologists (FN-01; ESU-01). Additionally, after the Syrian displacement, VPD indicators such as AFP incidence received attention and funding that required continuous collaboration between the EPI partners [35] (FN-01; ESU-01).

Despite successful coordination with EPI, the ESU’s coordination with the Primary Healthcare department (PHC) appeared limited (FN-01; ESU-03). The ESU reported collaboration with peripheral PHC facilities, but not centrally (FN-01; ESU-03). The MOPH PHC oversaw both public and private primary healthcare in Lebanon [32], providing first-line healthcare for refugees since the Syrian conflict began (MOPH-03, UN-02), and thus effective coordination between the two programmes could potentially improve infectious disease reporting (FN-01). Communication between the ESU and the MOPH preventive medicine service, responsible for prevention and control, was similarly limited (FN-01; ESU-03, NGO-03). Communication and information sharing was encouraged through initiation of weekly MOPH meetings, but two interviewees described these as relatively ineffectual (ESU-03, NGO-03).

Government hospitals were another major infectious disease surveillance hub according to official documentation [1] (Law number 544, article 3, 1996). Most ESU referral laboratories were in government hospitals and were important ESU stakeholders (ESU-03). However, many interviewees described the relationship as lacking clear operating procedures and TORs (FN-01; ESU-02, GH01). The ESU interviewees reflected that there should be no difference in infectious disease reporting between private and public facilities (ESU-03), though government facilities with high staff turnover required continuous reminding about reporting procedures (ESU-01, NGO-03).

#### Stakeholder coordination and communication

ESU’s slow engagement in the refugee crisis, as noted previously, meant many stakeholders did not know of its existence and unintentionally advocated duplicating surveillance efforts (ESU-02, NGO-03). Three factors that may have contributed to collaboration gaps were described across interview, observation, and document data. First, UN agencies working in Lebanon with the ESU did not mention its existence to the international community during health working-group meetings. Second, newly arriving UN agencies and INGOs did not attempt to understand the structure of Lebanese ministries and resources already in place before initiating their own responses (NGO-03). Finally, high turnover of international staff working with refugees meant many were not informed about ESU’s work and continued reminders were necessary (ESU-01, NGO-03). However, site visits helped identify two divergent accounts of positive collaboration at provincial level. First, an INGO interviewee in central Bekaa described good communication with the ESU team, easy contact with the ESU, and that the ESU was very responsive, particularly during a diarrhoea outbreak in which NGO staff were trained on testing and maintained good cooperation (NGO-01). Second, a health facility interviewee on the Syrian border described how effective collaboration and reporting to the ESU began after attending the health working group meeting (ESU-02, MC-01): “[Now, it’s] excellent, we’re in contact through the phone, we don’t even wait for emails and faxes” MC-01.

An example of stakeholder contributions to the ESU surveillance were Mobile Medical Units (MMU) and Outreach Volunteers (OVs)—established and funded by INGOs and some UN agencies to support Syrian refugees. The MMU helped the ESU access infectious disease data from Syrians in informal tented settlements (UN-01, NGO-01, ESU-02) and were added to the ESU’s medical centre surveillance system (ESU-01, ESU-03, NGO-01; Invitation Letter to ESU training sessions, 2013–2017; ESU-02, ESU-03). Funders described MMUs as efficient and timely in identifying and reacting to any potential outbreak among Syrian refugees (NGO-01, UN-01). OVs were recruited from Syrian refugee populations to help identify needs and rumours about infectious diseases among refugee populations, especially relating to high-risk diseases such as AFP and measles (ESU-01, ESU-03, UN-01, NGO-01).

UNRWA communication with the ESU appeared inconsistent for surveillance targeting Palestinian refugees (FN-03), potentially due to recent staffing changes. UNRWA health-worker interviewees were unaware of MOPH surveillance yet expressed interest in attending national trainings and strengthening collaboration (UN-03, MC-02). Conversely, the ESU staff were aware of UNRWA’s parallel system and noted information sharing had previously been regular and helpful (UN-03, MC-02, ESU-03): "[UNRWA] have a system that is working not only in Lebanon but also in other countries" (ESU-03).

A private-sector researcher described how conducting research was challenging because of public sector bureaucracy and collaboration challenges with ESU, with grants sometimes lost due to slow MOPH decision-making (RA-01), while lack of acknowledgement for information they shared could hurt academic’s careers (RA-01). Others similarly described a lack of feedback after reporting to ESU (MC-01, RA-01): "I never received any feedback about any changes done, to be honest…” (MC-01).

The ESU information dissemination for action was considered successful by interviewees, who attributed the lack of alarming infectious disease case numbers to good surveillance (ESU-01-03, NGO-03, RA-02). The ESU disseminated information through several channels. The ESU webpage provided public information on infectious diseases, electronic SOPs, and guidelines on reporting forms (FN-01; ESU-03). Other information was provided on request to the DG or presented in the MOPH meetings or the UNHCR-led meetings and trainings (FN-01, FN-02; ESU-02, ESU-03). ESU-03 described her study findings, on vaccine efficacy during a mumps outbreak among Palestinian refugees, as influencing UNRWA’s decision to start administering two MMR doses rather than one (ESU-03). ESU-02 noted that at the start of a leishmaniasis outbreak, data led to opening treatment centres near the residences of most cases rather than having them travel to distant locations (ESU-02).

## Discussion

### Key findings and implications

This study is a first effort to examine socio-political and organisational influences on the development and practice of infectious disease surveillance for refugees in Lebanon. The most important political influence related to Lebanon’s government not signing the 1951 Refugee Convention [2,3], thus avoiding a set of obligations towards refugees and responding through hospitality norms [2]. The concept of hospitality (‘karam’ in Arabic), also seen in Syria, Lebanon, and Turkey, relied on social norms of generosity to guests rather than international mandates for accepting refugees [28]. Despite international efforts to have Lebanon sign the Convention, ongoing political turmoil made it a low political priority [2]. This likely contributed to delayed government and ESU responses to Syrian forced displacement into Lebanon [5,29], which consequently reduced the visibility of the ESU’s surveillance work. For example, Boustani *et al* [40] noted that due to Lebanon’s stance on refugees, international organisations had to adjust their action plans pragmatically over time and ‘work with what was available’ [40].

A second political issue, related to ‘karam’ and Lebanon’s stance of refugees as guests [28], was the lack of dedicated refugee infectious disease surveillance. The ESU did not establish separate surveillance at refugee sites, instead amending its system to include refugees in aggregated national figures. It is unclear whether Lebanon could have benefited from adding syndromic surveillance to routine surveillance, to help with early detection and response for epidemics affecting refugees, such as the Hepatitis A cluster [12,41,42].

The first reason identified for this lack of dedicated surveillance was the ESU’s ethos that surveillance should target all residents without discrimination. Though logical, this could be limiting, as government reference to refugees as ‘guests’ would have been challenged by establishing desegregated surveillance [2,28]. A second undiscussed obstacle may have been sustainability concerns, due to time and resource constraints, as found in Italy’s syndromic surveillance for migrants [43]. This lack of disaggregated surveillance for refugees contributed to the absence of representativeness, which is important for effective surveillance [12]. The WHO advocates using different reporting systems depending on the needs and representativeness of all populations [12]. Relying on routine surveillance for detecting refugee infectious diseases, without targeting them specifically, can arguably delay representation and lead to untimely or inadequate prevention [12].

Related to the slow government response, but of direct relevance to ESU decision-making, is whether the ESU’s slow response suggested absence of an autonomous organisational willingness or ability to respond to crises [10–12]. The ESU’s more proactive response after 2013 appears to be a result of motivated personnel and additional international support and funding. A lesson from this is that sufficient staff motivation and funding can improve organisational responsiveness. Additionally, successful work and collaborations appeared to rely on proactive individuals rather than strong organisational culture [44]. Developing a supportive organisational culture could help encourage team-based responses rather than relying on individual initiative during crises [45,46].

Collaboration successes could be built upon. Literature supports improved collaboration with partners and refugee communities, identified for example in Albania and Cote d’Ivoire, as the most important surveillance elements during crises [14,18,19,47]. Lebanon’s experience reflected this, as perceptions of the ESU improved over time as proactive central and provincial staff began working with external stakeholders. Despite these benefits and individual successes, we found no clear MOPH/ESU approaches or objectives for working with partners during crises and many examples of parallel initiatives (e.g. UNRWA). The ESU should increase its national profile and take the lead on all surveillance activities to reduce duplication and develop clear communication and collaboration with partners in any future crisis. The ESU leadership could develop an emergency preparedness plan—as part of strategic planning—to support prompt and proactive action as recommended by WHO [48].

### Limitations

Several limitations should be considered. First, a short six-month data collection period and refusal of two potential interviewees could have resulted in some loss of depth, as findings from their organisations then relied on document review and observation. Second, though not necessarily a limitation, data were collected by one researcher (MS) as part of doctoral studies. MS worked in ESU and was known to some study participants. This emic perspective could have influenced responses and interpretation, e.g. by interviewees being overly positive or closed/cryptic in responses. In fact, several seemed to use interviews as an opportunity to complain about the ESU. We reported responses as recorded, in keeping Braun & Clarke’s thematic approach, while ensuring critical self-reflection about preconceptions, relationship dynamics, and analytic focus through reflective journaling and discussion between co-authors [23,49]. Third, this study did not include refugee perspectives due to ethics restrictions and future research should ideally do so. Fourth, protecting anonymity within the small pool of key informants on infectious disease surveillance in Lebanon prevented our reporting relevant interviewee characteristics that could aid interpretation of their perspectives. Finally, this study does not investigate the surveillance approaches for displaced populations during COVID-19 response activities as research was conducted before the start of the pandemic. Thus, it would be useful for future research to identify whether any organisational or collaboration changes occurred since.

### Conclusions

We examined how socio-political, internal, and external, factors affected the ESU’s surveillance for refugees, finding no systematic approach to refugee surveillance. Though the ESU led surveillance nationally, some partners still conducted parallel activities. In addition to national politics, lack of a clear institutional hierarchy, motivated personnel at peripheral offices, or organisational culture, made surveillance work harder and there is scope for the ESU to improve its ability to respond to future infectious disease outbreaks affecting refugees. More literature from countries experiencing large refugee influxes is needed to better situate our findings within the currently inadequate evidence base on socio-political and organisational aspects of infectious disease surveillance for displaced populations [14].

## References

[1] Ammar W, Kdouh O, Hammoud R, Hamadeh R, Harb H, Ammar Z, et al. Health system resilience: Lebanon and the Syrian refugee crisis. Journal of Global Health. 2016;6(2). doi: 10.7189/jogh.06.020704 28154758 PMC5234495

[2] Janmyr M. No Country of Asylum: ‘Legitimizing’ Lebanon’s Rejection of the 1951 Refugee Convention. International Journal of Refugee Law. 2017;29(3):438–65.

[3] Human Rights Watch. Rot Here or Die There Bleak Choices for Iraqi Refugees in Lebanon. Human Rights Watch. 2007;19(8).

[4] Knudsen A. Widening the protection gap: The “politics of citizenship” for Palestinian refugees in Lebanon, 1948–2008. Journal of Refugee Studies. 2009;22(1):51–73.

[5] Frangieh G. Syrian Refugees in Limbo: Is Lebanon’s Establishment of Camps the Answer? Legal Agenda. 2014.

[6] UN General Assembly. Report of the Special Rapporteur on adequate housing as a component of the right to an adequate standard of living, and on the right to non-discrimination in this context [Internet]. Vol. 15, International Organization. 2018 p. 23. Available from: https://www.cambridge.org/core/product/identifier/S0020818300024796/type/journal_article.

[7] CAS. التعداد العام للسكان والمساكن في مخيمات والتجمعات الفلسطينية في لبنان - 2017 [Internet]. 2017. Available from: https://www.pcbs.gov.ps/Downloads/book2356.pdf.

[8] UNHCR. Operational data portal: Refugee situations [Internet]. 2022 [cited 2022 Jan 1]. Available from: https://data2.unhcr.org/en/situations/syria/location/71.

[9] Chaaban J, Salti N, Ghattas H, Irani A, Ismail T, Batlouni L. Survey on the Socioeconomic Status of Palestine Refugees in Lebanon 2015. 2015.

[10] Levy BS, Sidel VW. Documenting the Effects of Armed Conflict on Population Health. Annual Review of Public Health. 2016;37(1):205–18. doi: 10.1146/annurev-publhealth-032315-021913 26989827

[11] Elias CJ, Alexander BH, Sokly TAN. Infectious Disease Control in a Long-term Refugee Camp: The Role of Epidemiologic Surveillance and Investigation. 1990;80(7):824–8. 10.2105/ajph.80.7.824 PMC1404980 2356906

[12] World Health Organization. Communicable disease surveillance and response systems—Guide to monitoring and evaluating. 2006 p. 90.

[13] IFRC. Middle East and North Africa IFRC Country Office: Country Programme Overview 2019. 2019 p. 14.

[14] Saleh M, Farah Z, Howard N. Infectious disease surveillance for refugees at borders and in destination countries: a scoping review. BMC Public Health. 2022;1–14. 35114956 10.1186/s12889-022-12646-7 PMC8813574

[15] MOPH. Classical reporting system [Internet]. 2019. Available from: https://www.moph.gov.lb/en/Pages/0/11352/classical-reporting-system.

[16] Ammar W. Health System and Reform in Lebanon. Beirut: Entreprice Universitaire d’Etudes et de Publications; 2003.

[17] MOPH. Epidemiological Surveillance [Internet]. 2018 [cited 2018 Mar 7]. Available from: https://www.moph.gov.lb/en/Pages/2/193/esu.

[18] Bozorgmehr K, Samuilova M, Petrova-Benedict R, Girardi E, Piselli P, Kentikelenis A. Infectious disease health services for refugees and asylum seekers during a time of crisis: A scoping study of six European Union countries. Health Policy. 2018. doi: 10.1016/j.healthpol.2018.04.003 29673804

[19] Valenciano M, Pinto A, Coulombier D, Hashorva E, Murthi M. Surveillance of communicable diseases among the Kosovar refugees in Albania, April-June 1999. Euro Surveill. 1999;4(9). doi: 10.2807/esm.04.09.00079-en 12631890

[20] Yin RK. Case study research: design and methods. Vol. 5. 1994. 170 p.

[21] Hannes K. Chapter 4 –Critical appraisal of qualitative Key points. In: Noyes J, Booth A, Hannes K, Harden A, Harris J, Lewin S, et al., editors. Supplementary Guidance for Inclusion of Qualitative Research in Cochrane Systematic Reviews of Interventions. Cochrane Collaboration Qualitative Methods Group; 2011.

[22] Green J, Thorogood N. Qualitative Methods for Health Research. London: SAGE Publications Ltd; 2004. 440 p.

[23] Braun V, Clarke V. Using thematic analysis in psychology. Qualitative Research in Psychology. 2006;3(2):77–101.

[24] Maxwell JA. Qualitative Research Design: An interactive approach. Sage Publications, Inc.; 2013.

[25] Braun V, Clarke V, Braun V, Clarke V. To saturate or not to saturate? Questioning data saturation as a useful concept for thematic analysis and sample-size rationales useful concept for thematic analysis and sample-size rationales. Qualitative Research in Sport, Exercise and Health. 2021;13(2):201–16.

[26] Varvasovszky Z, Brugha R. How to do (or not to do)… A stakeholder analysis. Health Policy and Planning. 2000;15(3):338–45. 11012410 10.1093/heapol/15.3.338

[27] UNHCR. Syria Regional Refugee Response [Internet]. 2021. Available from: https://reporting.unhcr.org/lebanon.

[28] Chatty D. The duty to be generous (karam): Alternatives to rights-based asylum in the Middle East. Journal of the British Academy. 2017;5(October):177–99.

[29] Frangieh G, Barjas E. Interior Ministry Advisor: Lebanon Refugee Policy Based on Set of “Nos.” Legal Agenda. 2016.

[30] Blanchet K, Fouad FM, Pherali T. Syrian refugees in Lebanon: The search for universal health coverage. Conflict and Health. 2016;10(1):1–5. doi: 10.1186/s13031-016-0079-4 27252775 PMC4888673

[31] WHO, IOM, MOPH. Joint review of the National Tuberculosis Programme of Lebanon. 2015 p. 35.

[32] MOPH. The ministry of public health [Internet]. 2018 [cited 2019 Feb 3]. Available from: https://www.moph.gov.lb/en/DynamicPages/index/9/1024/the-ministry.

[33] MOPH. Notifiable communicable diseases: Syrian refugees [Internet]. 2022. Available from: https://www.moph.gov.lb/userfiles/files/Esu_data/Esu_currentyear/SYRNRF.htm.

[34] Alawieh A, Sabra Z, Langley EF, Bizri AR, Hamadeh R, Zaraket FA. Assessing the impact of the Lebanese National Polio Immunization Campaign using a population-based computational model. BMC Public Health. 2017;17(1):1–11. 29178859 10.1186/s12889-017-4909-0 PMC5702188

[35] WHO. Global Polio Surveillance Action Plan, 2018–2020. 2019 p. 86.

[36] Bizri A, Fares J, Musharrafieh U. Infectious diseases in the era of refugees: Hepatitis a outbreak in Lebanon. Avicenna Journal of Medicine. 2018;8(4):147. doi: 10.4103/ajm.AJM_130_18 30319956 PMC6178566

[37] Alawieh A, Musharrafieh U, Jaber A, Berry A, Ghosn N, Bizri AR. Revisiting leishmaniasis in the time of war: The Syrian conflict and the Lebanese outbreak. International Journal of Infectious Diseases. 2014;29:115–9. doi: 10.1016/j.ijid.2014.04.023 25449245

[38] Ozaras R, Leblebicioglu H, Sunbul M, Tabak F, Balkan II, Yemisen M, et al. The Syrian conflict and infectious diseases. Expert Review of Anti-Infective Therapy. 2016;14(6):547–55. doi: 10.1080/14787210.2016.1177457 27063349

[39] UNRWA Who we are. 2018.

[40] Boustani M, Carpi E, Gebara H, Mourad Y. Responding to the Syrian Crisis in Lebanon Collaboration between Aid Agencies and Local Governance Structures. Policy and planning; Urban. 2016.

[41] ECDC. Handbook on implementing syndromic surveillance in migrant reception / detention centres and other refugee settings. 2016.

[42] Henning KJ. Overview of Syndromic Surveillance What is Syndromic Surveillance? CDC MMWR. 2004;53. 15714620

[43] Napoli C, Riccardo F, Declich S, Dente MG, Pompa MG, Rizzo C, et al. An early warning system based on syndromic surveillance to detect potential health emergencies among migrants: Results of a two-year experience in Italy. International Journal of Environmental Research and Public Health. 2014;11(8):8529–41. doi: 10.3390/ijerph110808529 25140999 PMC4143875

[44] West MA, Lyubovnikova J. Illusions of team working in health care Illusions of team working in health care. Journal of Health Organization and Management. 2013;134–42. 23734481 10.1108/14777261311311843

[45] Scott T, Mannion R, Davies HTO, Marshall MN. Policy Roundtable Implementing culture change in health care: theory and practice. International Journal for Quality in Health Care. 2003;15(2):111–8. 12705704 10.1093/intqhc/mzg021

[46] O’Reilly CA, Chatman JA. Culture as social control: Corporations, cults, and commitment. Research in Organizational Behavior. 1996;18(June):157–200.

[47] Kouadio IK, Koffi AK, Attoh-Toure H, Kamigaki T, Oshitani H. Outbreak of Measles and Rubella in Refugee Transit Camps. Epidemiology and Infection. 2009;137(11):1593–601. doi: 10.1017/S0950268809002520 19379539

[48] WHO. A Strategic Framework for Emergency Preparedness. 2017;29.

[49] Galdas P. Revisiting Bias in Qualitative Research: Reflections on Its Relationship With Funding and Impact. International Journal of Qualitative Methods. 2017;16(1):1–2.

